# Salivary ultrasonography and histopathologic evaluation of secondary Sjögren’s syndrome in rheumatoid arthritis patients

**DOI:** 10.1038/s41598-023-38469-z

**Published:** 2023-07-13

**Authors:** Youngjae Park, Minae Oh, Youn Soo Lee, Wan-Uk Kim

**Affiliations:** 1 Division of Rheumatology, Department of Internal Medicine, Seoul St. Mary’s Hospital, College of Medicine, The Catholic University of Korea, Seoul, Korea; 2 Department of Hospital Pathology, Seoul St. Mary’s Hospital, College of Medicine, The Catholic University of Korea, Seoul, Korea; 3 Center for Integrative Rheumatoid Transcriptomics and Dynamics, The Catholic University of Korea, Seoul, Korea

**Keywords:** Medical research, Rheumatology

## Abstract

Novel modalities, such as salivary ultrasonography (SGUS) and shear wave elastography (SWE), have previously been introduced to evaluate Sjögren’s syndrome (SS). However, in secondary SS (sSS), the diagnostic performance of SGUS and its relationship with clinicopathological characteristics have not yet been clearly defined. In this study, we aimed to investigate sSS in RA patients using SGUS and SWE and sought to determine its pathological correlations. Thirty-one RA patients who presented with sicca symptoms were included to be evaluated on SS, and were compared with 18 primary SS (pSS) patients. All subjects were assessed through SGUS, SWE, and conventional diagnostic approaches for SS, including minor salivary gland biopsy (MSGB). In SGUS evaluation, two separate scoring systems, suggested by Hocevar and OMERACT, were used. Among 31 RA patients with sicca symptoms, 19 (61.2%) were diagnosed as sSS. Similar to pSS, SGUS showed good diagnostic performance (sensitivity 68.4% and 78.9%, and specificity 91.7% and 75.0% for Hocever and OMERACT, respectively) in differentiating sSS from RA patients with simple sicca symptoms. The sSS and pSS patients exhibited significantly higher lymphoid infiltration areas in MSGB than RA patients without SS. Focus score and lymphoid infiltration areas correlated well with sonographic severity. Severity of fibrosis in MSGB showed better positive correlation with SWE than with SGUS. Similar to pSS, SGUS shows good diagnostic performance for sSS in RA patients. SWE reflects histopathologic chronicity of MSGB well in both pSS and sSS.

## Introduction

Sjögren’s syndrome (SS) is an autoimmune disease that primarily presents with dry eyes and dry mouth that originate from inflammatory infiltration into exocrine glands^1^. This condition manifests both sicca symptoms, and various systemic involvements^2^. SS can influence affected patients as an isolated form, which is regarded as primary SS (pSS); whereas, as an overlapped form, it is designated as secondary SS (sSS), with other connective tissue diseases^3^. Rheumatoid arthritis (RA), which is a chronic autoimmune disease that mainly involves bone and joint, is one of the most frequently overlapped medical conditions with SS^4^. The two diseases, SS and RA, can interactively influence each other’s pathogenesis and disease activities^4,5^. Therefore, identifying these potentially overlapped autoimmune conditions is crucial for the affected patients to manage consequences arising from both diseases. However, despite the clinical significance, the prevalence of sSS in RA patients has to date not been precisely clarified.

According to a recently published classification criteria for SS announced by the American College of Rheumatology (ACR) and the European League Against Rheumatism (EULAR) in 2016, diagnosis of SS can be made based on the prevalence of anti-Ro antibodies in patients’ sera and the evaluation of minor salivary gland biopsy (MSGB)^6^. However, considering the inconvenience of MSGB and the poor availability of measuring anti-Ro antibodies, several novel diagnostic modalities using imaging devices have recently been attempted to determine the diagnosis of SS. Salivary gland ultrasonography (SGUS) is one of the most promising modalities for diagnosing SS with its ease of application and wide availability^7^. Multiple previous studies reported that SGUS can confer fair diagnostic performance by itself, or replace one of the items in the 2016 classification criteria for SS as an alternative item^7–9^. Furthermore, beyond its usefulness as a diagnostic tool, a recently published study suggested that SGUS can reflect the degree of inflammatory infiltrates in major salivary glands, which represents the severity of autoimmunity in the exocrine glands of SS patients^10^.

In addition to SGUS, shear wave elastography (SWE) is also suggested as an alternative tool to assess the severity of several medical conditions, including SS^11^. SWE can be used to measure the elasticity of tissues. Values acquired from SWE can indicate the stiffness of tissues, thereby representing the severity of fibrosis, according to the previous reports^12^. Because advanced state of SS progression can result in the fibrotic replacement of normal glandular tissues, SWE can assess the degree of fibrosis in major salivary glands, and can be expected to predict the reversibility of the disease and the efficacies of cholinergic agonists in SS patients^13^.

Although various studies reported the usefulness of SGUS and SWE in pSS, the diagnostic performance of these novel modalities and their relationships with clinicopathologic features have scarcely been explored in sSS^7,9–12^. Here, considering the clinical significance of sSS in RA, we sought to determine the diagnostic performance of SGUS in sSS, and the correlations between the findings of emerging imaging modalities and the key pathological features of SS.

## Materials and methods

### Targeted population and diagnostic tests for Sjögren’s syndrome

We included RA patients with sicca symptoms at least for more than 1 month who fulfilled the 2010 ACR/EULAR classification criteria for RA as targeted subjects, and pSS patients who fulfilled the 2016 ACR/EULAR classification criteria for SS as controls^6,14^. All subjects were followed up in the rheumatologic department of Seoul St. Mary’s hospital, a tertiary care university-affiliated hospital and referral center in Seoul, Korea and enrolled in the present study between April 2021 and March 2022. Among RA patients, we excluded subjects who were already diagnosed as Sjögren’s syndrome before the study enrolment, or who were overlapped by other connective tissue diseases, such as lupus and scleroderma, or medical conditions, such as previous radiation history to the head and neck area, lymphoproliferative diseases, and graft-versus-host disease. All subjects, including RA patients and pSS patients, were fully assessed for diagnostic approaches for SS, including laboratory tests, salivary flow rate measurement, Schirmer I test, ocular staining score, and MSGB. Disease activity indices for RA and SS were measured by disease activity score 28 (DAS28) and EULAR Sjögren’s syndrome disease activity index (ESSDAI), respectively^15,16^. Simultaneously, we performed SGUS and SWE on the major salivary glands of all targeted patients, as below. All methods in this study were performed in accordance with the Declaration of Helsinki.

### Histopathologic evaluation of minor salivary gland biopsy

**Figure 1:**
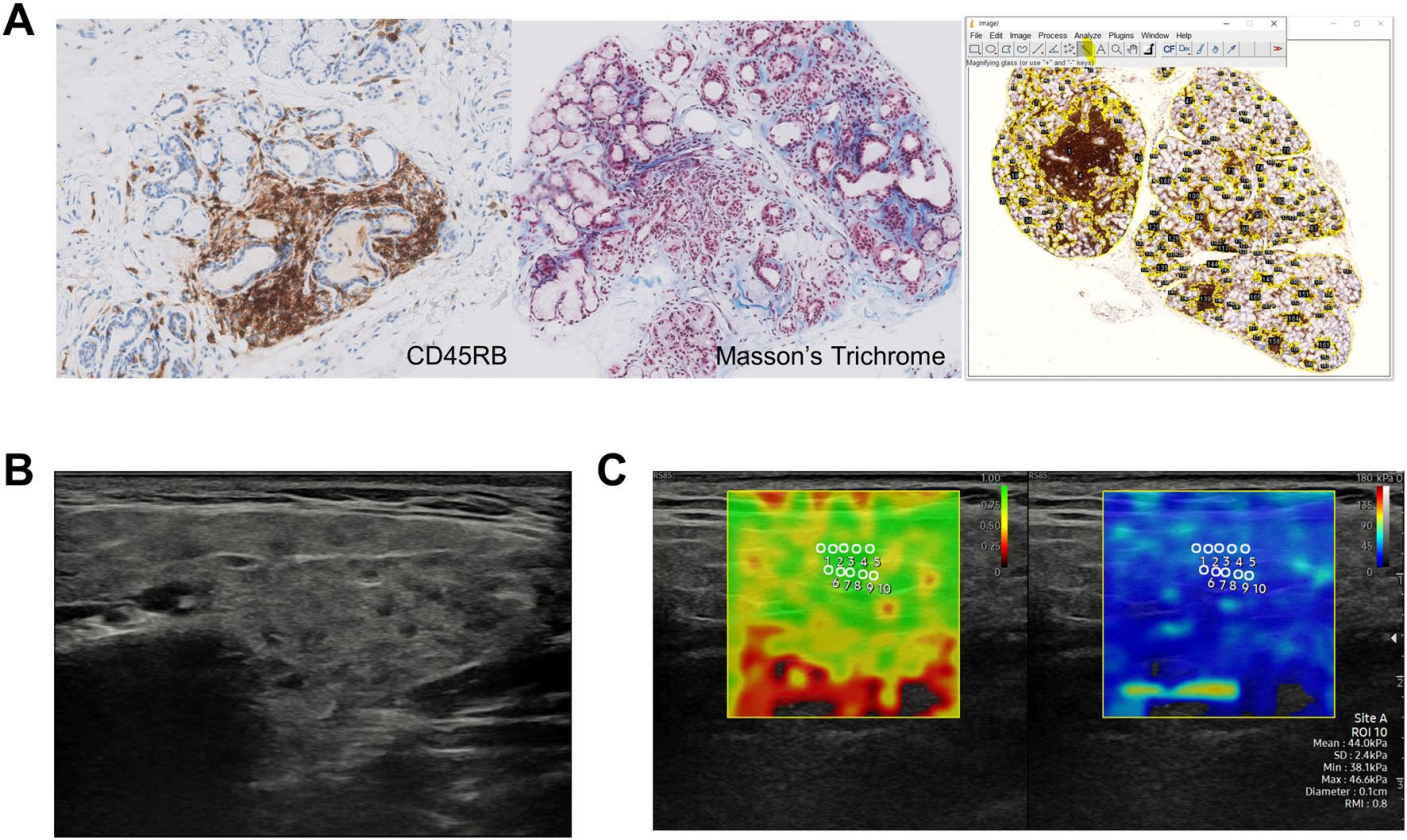
Representative images of histopathologic evaluation of minor salivary gland biopsy, salivary gland ultrasonography, and shear wave elastography. (**A**) Representative histologic images of minor salivary gland biopsy specimens stained with CD45RB and Masson’s Trichrome, and an example of calculation of the relative proportion of targeted area among overall specimens. Original magnification × 100. (**B**) A representative image of salivary gland ultrasonography in a parotid gland of a patient with Sjögren’s syndrome. (**C**) A representative image of shear wave elastography in a parotid gland of a patient with Sjögren’s syndrome.

MSGBs were performed in all targeted subjects at the time of study enrolment. All biopsy procedures and calculation of focus score, which represents lymphocytic infiltration in salivary glands, were performed according to the SICCA protocols^17^. To define the severity of inflammatory infiltration and fibrosis in MSGB, we performed immunohistochemical staining using anti-CD45RB antibody and special staining using Masson’s trichrome (MT), respectively. All the histologic slides acquired from MSGB were digitally scanned, and percentages of area with inflammation (anti-CD45RB-stained) and fibrosis (blue-colored area in MT staining) among overall glandular tissues were calculated using Image J version 1.8 by an experienced rheumatologist (MO) and a pathologist (YSL) (Fig. 1A).

### Salivary gland ultrasonography

Ultrasonographic scanning was done by two experienced rheumatologists (YP and MO) using a RS85A ultrasound scanner (Samsung Medison Co., Seoul, Korea) equipped with a broad-spectrum probe of 5–12 MHz. Two independent scoring systems for diagnosing SS, one suggested by Hocevar et al., and the other by the outcome measures in rheumatology (OMERACT), were adopted in the present study^18,19^. Both scoring systems showed good diagnostic performance and validities in the previous studies for pSS^20,21^. We measured these scores in all four major salivary glands, including right and left parotid glands and submandibular glands, and summed them up into total scores for each patient (Fig. 1B). Sonographic positivity for SS was defined for a total score ≥ 17 for Hocevar’s method, while for a grade ≥ 2 in at least one gland in the OMERACT method^18,22^.

### Shear wave elastography

SWE was performed by the rheumatologist (MO) above using the same scanner machine equipped with a shear wave probe. We selected at least 10 regions of interest that were at least 2 mm apart from the glandular surfaces in each major salivary gland (Fig. 1C), measured their elasticity in units of kilopascal (kPa), and then calculated the averaged elasticities in each parotid and submandibular gland of separate subjects.

### Statistical analysis

Continuous variables were analyzed with the Mann–Whitney *U* test and expressed as medians and interquartile ranges in all the table and figures. Categorical variables were analyzed with the Chi-squared test and Fisher’s exact test. The degree of relationship between two independent variables was analyzed by the Spearman’s rank correlation coefficient test. All statistical analyses were performed using IBM-SPSS Statistics version 24.0 (SPSS Inc., Chicago, IL, USA). All figures were drawn using GraphPad Prism version 8.0 (GraphPad Software, San Diego, CA, USA). Statistical significance was considered if the *p* value was < 0.05.

### Ethics approval

This study was approved by the Institutional Review Board of Seoul St. Mary’s Hospital of the Catholic University of Korea (approval number: KC20OASE0631).

### Consent to participate

Informed consent was obtained from all individual participants included in the study.

## Results

### Baseline demographics, laboratory findings, and clinical indices

Among 31 RA patients who were evaluated for the presence of SS on the basis of sicca symptoms, 19 patients (61.2%) were finally diagnosed as sSS according to the 2016 ACR/EULAR classification criteria, while the other 12 patients were not. Most (93.5%) of the RA subjects were women (Table 1). The median ages of the three groups of ‘RA only’, ‘RA with sSS’, and ‘pSS’ were similar (Table 1). The median disease durations were also similar among the three groups. The median duration of sicca symptoms was longer in the ‘pSS’ group compared to the other groups although the difference was not statistically significant. Results of objective tests, including unstimulated salivary flow rates, Schirmer I test, and ocular staining score, for assessing the severity of xerostomia and xerophthalmia, were not different between the three groups. Interestingly, the positivity for anti-Ro antibodies and anti-La antibodies in patients’ sera were significantly higher in the ‘pSS’ group, compared to the ‘RA with sSS’ group (anti-Ro antibody positivity: 100% vs. 63.2%, *p* = 0.008; anti-La antibody positivity: 55.6% vs. 15.8%, *p* = 0.017) (Table 1). All patients’ sera were negative for anti-centromere antibodies. Other laboratory values and disease activity indices related to RA or SS, such as blood cell counts, serum levels of complements and immunoglobulins, DAS28, and ESSDAI, showed no significant intergroup differences.

**Table 1:** Clinical and laboratory variables related to Sjögren’s syndrome and rheumatoid arthritis.

	RA, n = 31		Primary SS, n = 18	*p* value^†^
	Without secondary SS, n = 12	With secondary SS, n = 19		
Age, years	66 (47–71)	57 (49–63)	57 (46–63)	0.831
Female	12 (100)	17 (89.5)	18 (100)	0.486
Disease duration, months	101 (12–195)	40 (2–159)	36 (4–72)	0.394^‡^
Sicca symptoms duration, months	7 (2–18)	4 (1–25)	59 (7–94)	0.083
Unstimulated salivary flow rate ≤ 1.5 mL in 15 min	8 (66.7)	14 (73.7)	14/17 (82.4)	0.695
Schirmer I test ≤ 5 mL/5 min	6/8 (75.0)	10/14 (71.4)	6/11 (54.5)	0.434
Ocular staining score ≥ 5	4/8 (50.0)	8/14 (57.1)	5/12 (41.7)	0.695
Anti-Ro antibody positivity	0 (0)	12 (63.2)	18 (100)	0.008
Anti-La antibody positivity	1/12 (8.3)	3 (15.8)	10 (55.6)	0.017
Minor salivary gland biopsy positivity	0 (0)	16 (84.2)	13 (72.2)	0.447
Focus score	0	1.8 (1–2.5)	3.0 (1–4.2)	0.285
Leukopenia (< 4.00 × 10^3^/mm^3^)	1 (8.3)	3 (15.8)	6 (33.3)	0.269
Neutropenia (< 1.50 × 10^3^/mm^3^)	0 (0)	1 (5.3)	4 (22.2)	0.180
Anemia (< 12 g/dL)	1 (8.3)	4 (21.1)	4 (22.2)	> 0.999
Thrombocytopenia (< 150 × 10^3^/mm^3^)	0 (0)	0 (0)	3 (16.7)	0.105
Anti-nuclear antibody positivity (titer ≥ 1:80)	8 (66.7)	17 (89.5)	18 (100)	0.486
Rheumatoid factor positivity (≥ 20 IU/mL)	10 (83.3)	17 (89.5)	4 (22.2)	0.630^‡^
Anti-CCP antibody positivity	8 (72.7)	18 (94.7)	0 (0)	0.126^‡^
Hypergammaglobulinemia	0 (0)	5 (26.3)	8 (44.4)	0.248
Immunoglobulin G, mg/dL	1145 (840–1372)	1352 (1145–1970)	1438 (1231–2106)	0.423
Low C3 (< 76 mg/dL)	0 (0)	1 (5.3)	0 (0)	> 0.999
Low C4 (< 12 mg/dL)	0 (0)	4 (21.1)	0 (0)	0.105
ESSDAI		3 (1–6)	2 (0–3)	0.141
DAS28	2.30 (1.43–2.78)	2.16 (1.33–4.20)		0.715^‡^
Hocevar score positivity	0 (0)	8 (42.1)	8 (44.4)	0.012^‡^
OMERACT score positivity	0 (0)	15 (78.9)	9 (50.0)	< 0.001^‡^

All data are presented as n (%) or median (interquartile range).

*RA* rheumatoid arthritis, *SS* Sjögren’s syndrome, *CCP* cyclic citrullinated peptide, *ESSDAI* EULAR Sjögren’s syndrome disease activity index, *DAS28* disease activity score 28, *OMERACT* the outcome measures in rheumatology.

^†^Values are calculated from comparison between the ‘RA with secondary SS’ group and the ‘primary SS’ group.

^‡^A value is calculated from comparison between the ‘RA without secondary SS’ group and the ‘RA with secondary SS’ group.

### SGUS scores in pSS and RA with sSS

**Figure 2:**
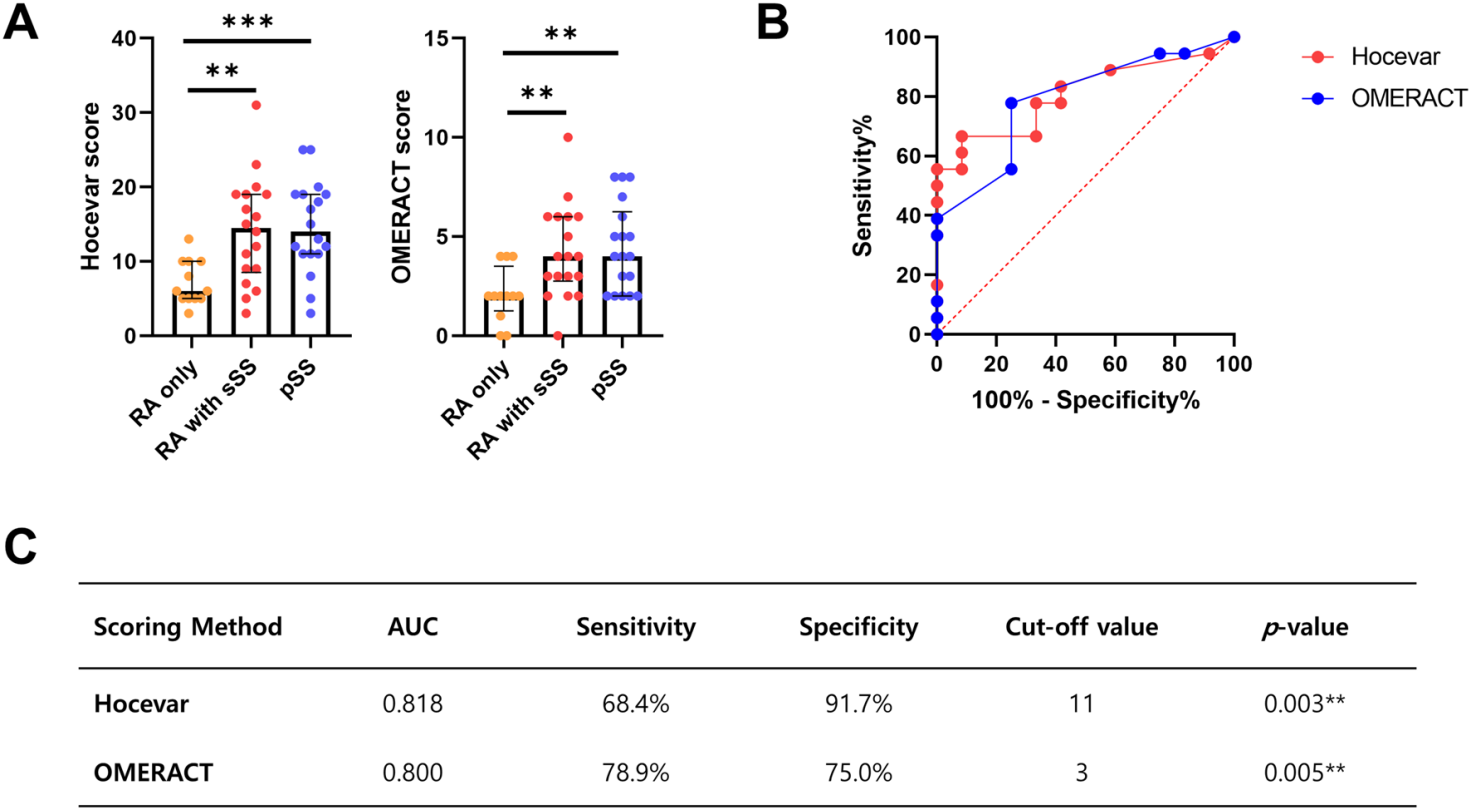
Two independent scoring systems of salivary gland ultrasonography and their diagnostic performance in diagnosing secondary Sjögren’s syndrome in rheumatoid arthritis patients. (**A**) Hocevar and OMERACT scores in primary and secondary Sjögren’s syndrome. (**B**) Receiver operating characteristic curves of Hocevar (a red line) and OMERACT (a blue line) in diagnosing secondary Sjögren’s syndrome in rheumatoid arthritis patients. (**C**) Area under the curves (AUC), sensitivity, specificity, and cut-off values of Hocevar and OMERACT in determining secondary Sjögren’s syndrome in rheumatoid arthritis patients. **p < 0.01, ***p < 0.001.

The positivity rates of SGUS scores according to both scoring systems, the Hocevar method and the OMERACT method, were significantly higher in the ‘RA with sSS’ group than in the ‘RA only’ group (the Hocevar method: 42.1% vs. 0%, *p* = 0.012; the OMERACT method: 78.9% vs. 0%, *p* < 0.001), as shown in Table 1. Absolute scores calculated by both scoring methods were also significantly increased in the ‘RA with sSS’ group and the ‘pSS’ group compared to the ‘RA only’ group (Fig. 2A). Intergroup differences of SGUS scores between the ‘RA with sSS’ group and the ‘pSS’ group were not significant.

The distinguishing power of SGUS for ‘RA with sSS’ from ‘RA without sSS’ could be determined by the receiver operating characteristic curves. As shown in Fig. 2B,C, SGUS assessed by the Hocevar method and the OMERACT method showed good performance for diagnosing sSS among RA patients with sicca symptoms with area under the curve values of 0.818 and 0.800, respectively. The sensitivity and specificity of the Hocevar method were 68.4% and 91.7%, respectively, while those of the OMERACT were 78.9% and 75.0%, respectively. From these findings, we assume that SGUS can be used as one of the diagnostic tools to determine sSS among RA patients.

### Relation between the degree of inflammatory infiltration in MSGB and SGUS scores

**Figure 3:**
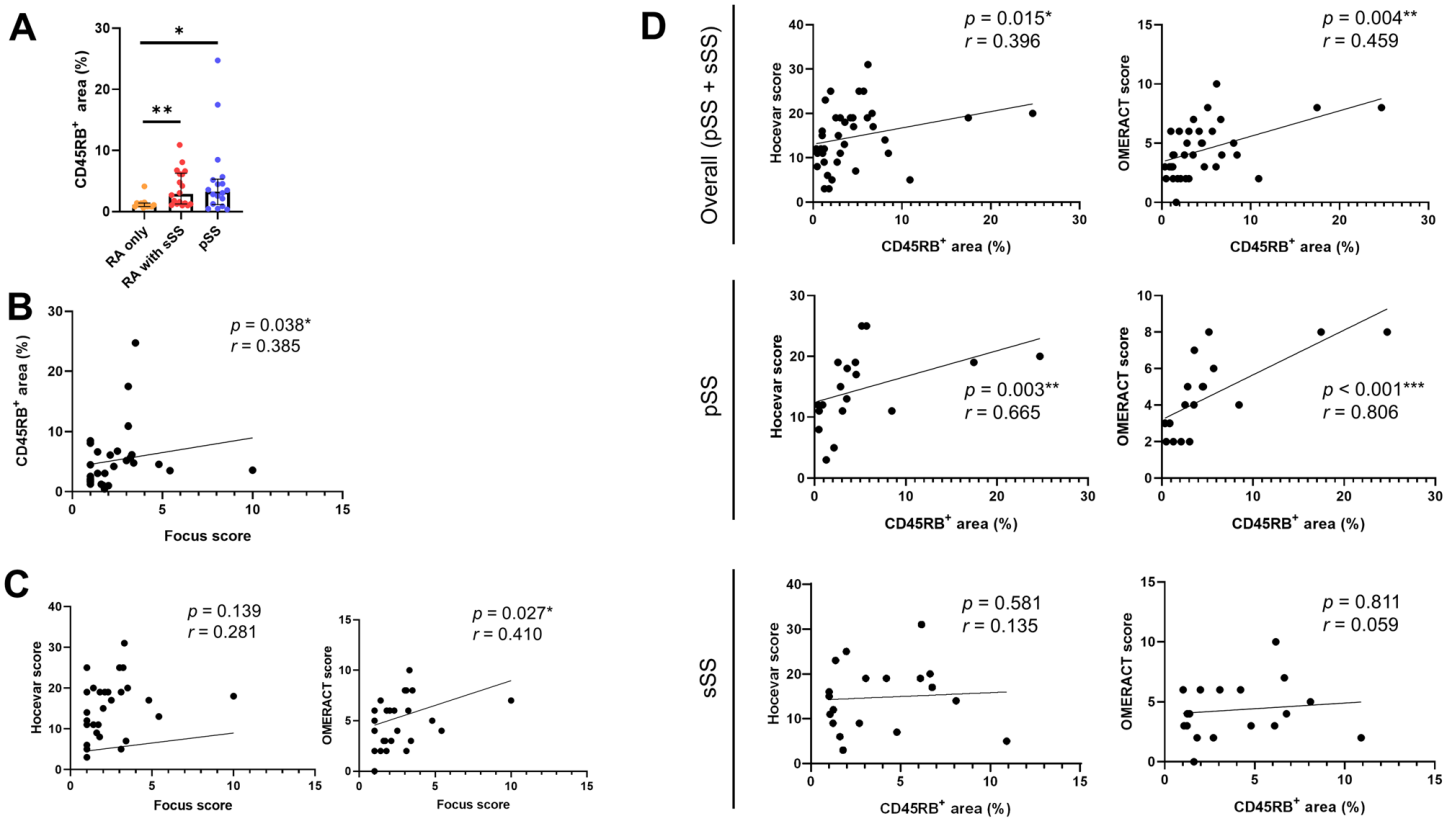
Relation between salivary gland ultrasonography (SGUS) and inflammatory infiltration in minor salivary gland biopsy (MSGB). (**A**) CD45RB-positive area of MSGB specimens in primary and secondary Sjögren’s syndrome. (**B**) Correlation between CD45RB-positive area in MSGB and focus scores in Sjögren’s syndrome. (**C**) Correlations between focus scores and SGUS scores (Hocevar and OMERACT method) in Sjögren’s syndrome. (**D**) Correlations between CD45RB-positive area in MSGB and SGUS scores (Hocevar and OMERACT method) of overall Sjögren’s syndrome, primary and secondary Sjögren’s syndrome. *p < 0.05, **p < 0.01, ***p < 0.001.

CD45RB has been used for the marker for most hematopoietic cells including lymphocytes in pSS^23^. Because lymphoid infiltration into exocrine glands is the pathognomonic feature in SS, we determined the degree of prevalence of CD45RB-positive cells in MSGB using immunohistochemistry. As shown in Fig. 3A, the relative areas (%) covered by CD45RB-positive cells were significantly increased in MSGB specimens from the ‘RA with sSS’ group and the ‘pSS’ group compared to those from the ‘RA only’ group. The relative areas of CD45RB-positive cells were positively correlated with focus score, the representative indicator of the pathologic severity of SS^24^, in pSS and RA with sSS patients (n = 29) (Fig. 3B).

We also investigated histopathologic features in the MSGB between pSS and sSS. The focus scores of MSGBs in two groups, pSS and sSS (with RA), showed no statistical differences (Table 1). The relative areas representing the degree of inflammatory infiltration and fibrotic changes in MSGBs were also similar between the two groups (Fig. 3A, and data not shown). In addition, we performed histopathologic comparison of MSGB specimens in terms of patterns of distorted structures that were potentially due to inflammation in the two groups (performed by YSL). However, as in the quantitative analyses, there were no distinct histopathologic features that distinguished sSS from pSS in MSGBs.

In comparison between the pathologic and sonographic severity, SGUS scores by the OMERACT method were well correlated with the focus score in MSGB specimens from SS patients (pSS + sSS patients), whereas those by the Hocevar method showed no statistical significance (Fig. 3C). On the other hand, the relative areas of CD45RB-positive cells in MSGBs from SS patients (pSS + sSS patients) presented with excellent correlation with both SGUS scoring systems (Hocevar: *p* = 0.015, OMERACT: *p* = 0.004) as seen in Fig. 3D (the upper panels**)**. These findings suggest that the SGUS scores in major salivary glands reflect well the inflammatory cell infiltration in MSGBs, and thus the pathologic severity in SS patients. However, such strong correlations were only observed in pSS (the middle panels of Fig. 3D) whereas those were absent in sSS (the lower panels of Fig. 3D).

### Relation between the degree of fibrosis in MSGB and SWE

**Figure 4:**
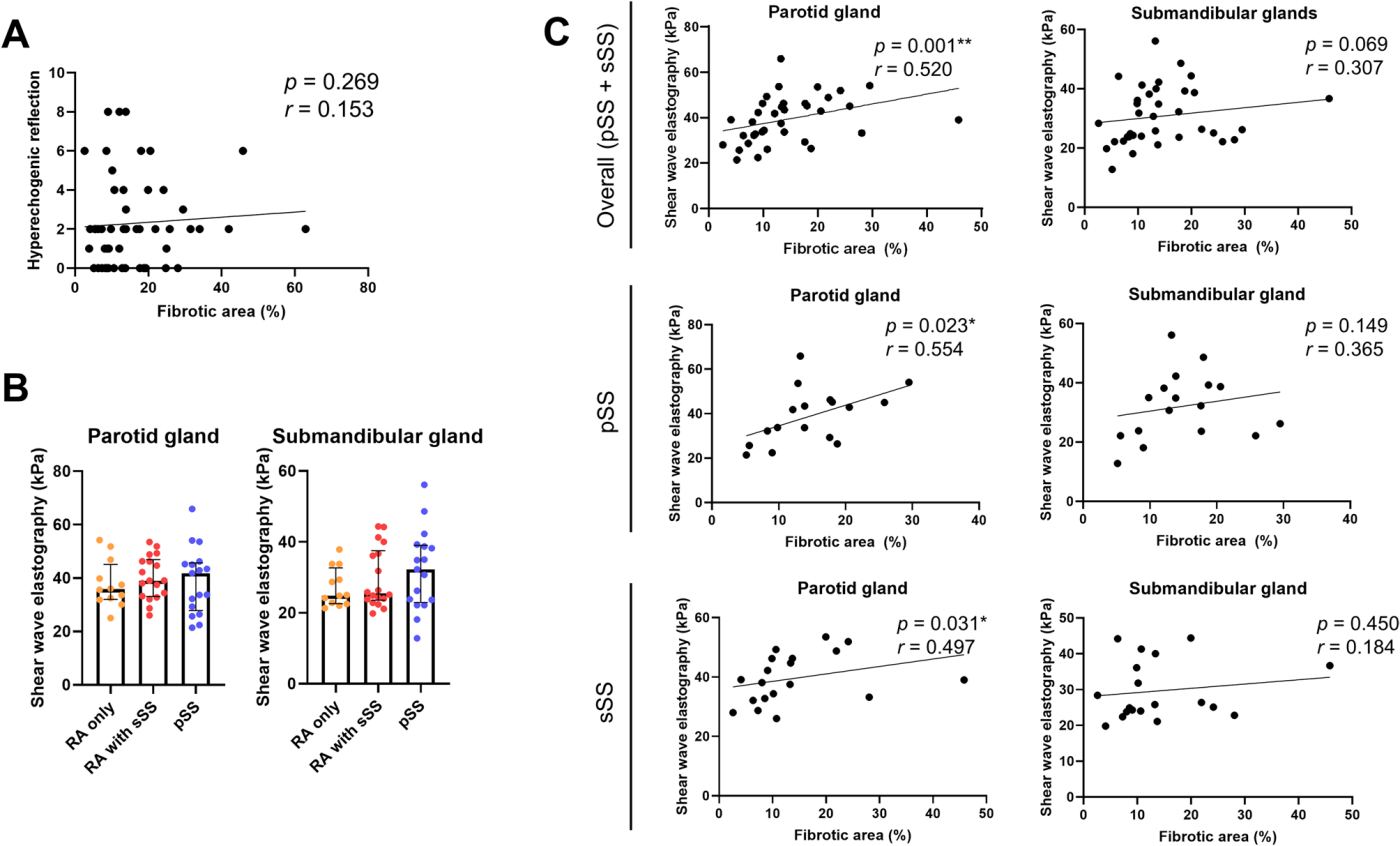
Relation between shear wave elastography (SWE) in major salivary glands and fibrosis in minor salivary gland biopsy (MSGB). (**A**) Correlation between hyperechogenic reflection scores of salivary gland ultrasonography measured by the Hocevar method and fibrotic area in MSGB. (**B**) SWE in major salivary glands of primary and secondary Sjögren’s syndrome patients. (**C**) Correlations between SWE in major salivary glands and fibrotic area in MSGB of overall Sjögren’s syndrome, primary and secondary Sjögren’s syndrome. *p < 0.05, **p < 0.01.

Long-term pathologic consequences of SS can result in irreversible fibrotic replacement of normal salivary gland tissues^13^. In such fibrotic condition, current medications relieving sicca symptoms may be ineffective. Therefore, we aimed to determine the usability of SWE in predicting the degree of fibrosis in MSGB specimens from SS patients. The degree of fibrosis was assessed by special staining with MT in MSGB specimens. In MT staining, collagen is stained with blue, representing the fibrotic changes of glandular structures. Because the previous study suggested that ‘hyperechogenic reflection’, which is one of sub-items in the Hocevar method for SGUS, represented the fibrosis of major salivary glands, we assessed relationship between this score and fibrotic areas in MSGB^10^. However, these two variables showed no significant correlation (Fig. 4A). The values of SWE measured in both parotid gland (median values: 41.8 kPa for pSS vs*.* 39.1 kPa and 35.8 kPa for sSS and RA only, respectively) and submandibular gland (median values: 32.3 kPa for pSS vs*.* 25.5 kPa and 24.9 kPa for sSS and RA only, respectively) tended to be higher in pSS patients than in the other two groups, but the difference was not statistically significant (Fig. 4B). Unexpectedly, we observed that the values of SWE in major salivary glands, especially parotid glands, showed correlations with the degree of fibrosis in MSGBs, represented by blue-stained areas in MSGBs stained with MT in both pSS and sSS (Fig. 4C). Collectively, SWE showed fair positive correlation with the fibrotic areas in MSGBs of SS, whereas hyperechogenic reflection using SGUS scores failed to show such association.

## Discussion

In this study, we demonstrated the diagnostic performance of SGUS in differentiating sSS among RA patients with sicca symptoms; even single measurement of SGUS performed according to two previously validated independent scoring systems presented with good sensitivity and specificity in diagnosing sSS. In addition, the SGUS scores of major salivary glands calculated by the two scoring methods correlated well with the severity of inflammatory infiltration in MSGB specimens from SS patients. Moreover, the elasticity values measured by SWE in parotid glands were significantly associated with the extent of fibrosis in MSGB.

The prevalence of sSS in RA patients has been reported as around 5–30%^25,26^. Wide ranges of reported prevalence may originate from the heterogeneity of targeted populations and difference of adopted classification criteria in each study. We observed 19 sSS cases among RA patients with relatively higher prevalence than those from other studies. Although we primarily focused on patients presenting sicca symptoms, we can assume, based on the present data, that there may be more subclinical or underdiagnosed sSS patients among the RA population than previously reported. A study regarding a large cohort for RA reported that RA with SS manifests higher disease activities than RA without SS^25^. However, our study showed no intergroup differences between RA with SS and RA without SS. Interestingly, sSS patients combined with RA presented with less prevalence of autoantibodies including anti-Ro antibodies and anti-La antibodies, in sera, compared to pSS patients. These results are consistent with other reports comparing the clinical features between sSS and pSS^27^. Considering these findings, we need to perform further evaluations such as MSGB or SGUS in RA patients with dry symptoms even if they are negative for anti-Ro/La antibodies in their sera.

The diagnostic performance of SGUS in pSS was reported to show the pooled sensitivity of about 70% and pooled specificity of about 90%, according to systematic reviews^7,28^. In our study, SGUS exerted compatible diagnostic powers with 68% of sensitivity and 91% of specificity by the Hocevar method in distinguishing sSS among RA patients, compared to those in pSS patients, although the more simplified scoring method, the OMERACT method, presented with relatively lower specificity, with 75%. Recent studies attempted to increase the diagnostic powers of previously announced classification criteria for pSS by adding SGUS as another item, or replacing pre-existing items with SGUS. According to these reports, SGUS can increase the sensitivity of classification criteria by about 5–20%^9,29–31^. Our results support that SGUS can also be performed as an additional diagnostic modality in determining whether sSS exists in certain subjects, as it does in pSS.

In addition to a role as a diagnostic tool, assessment of disease severity in respect of autoimmunity in targeted tissues, such as salivary glands, can be performed using SGUS in SS. A previous study reported fair associations between the focus scores and SGUS scores of labial glands in pSS^32^. Mossel et al. recently compared the histopathology of parotid glands and parotid gland ultrasonography, and demonstrated that inflammation represented by the percentages of CD45-positive areas in the parotid glands of pSS patients was better associated with the SS-related sonographic findings than was the focus score^10^. Consistent with these findings in pSS, our study showed good correlations between CD45RB-positive cell infiltrations in MSGB specimens, and SGUS scores in overall SS (pSS + sSS). We also observed that CD45RB-positive areas in MSGB were more significantly associated with SGUS scores than with focus score, as in other studies^10^. However, in the present study, such strong correlations between degrees of lymphocytic cell infiltration and SGUS scores were only observed in pSS, not in sSS. The potential reasons for this can include relatively shorter symptom durations and lower degrees of lymphocytic cell infiltration in sSS compared to pSS. Further studies performed in larger populations with similar symptom durations may address the raising question in this study. Overall, the SGUS scores of SS may represent CD45RB-positive lymphocytic cell infiltration in salivary glands especially in pSS.

According to a recent study, SS patients, especially with high focus scores, can present with increased fibrosis in minor salivary glands^13^. Therefore, fibrotic changes of salivary glands are regarded as advanced and progressed state of SS. In this study, a SGUS scoring item of hyperechogenic reflection indicating fibrotic changes did not show significant relationship with the pathologic severity of fibrosis in minor salivary glands. Rather, SWE was suggested as a potential alternative modality representing fibrosis in our study. Previous studies have reported that SWE shows good performance in reflecting SGUS scores, ESSDAI, and glandular dysfunctions^11,12,33^. Moreover, in other connective tissue diseases, such as scleroderma, SWE values also are substantially associated with the severity of tissue fibrosis^34,35^. Intriguingly, our data revealed that SWE values in major salivary gland (parotid glands) were fairly correlated with the extent of pathologic fibrosis in minor salivary glands, which is consistent with earlier reports^35^. If validation can be performed in further studies, SWE is expected to be applied to assess the fibrotic state of salivary glands in SS.

This study has some limitations. First, the numbers of overall subjects may be too small to reach statistical powers and reduce potential bias in some analyses. This limitation was due to methodologic difficulties from the simultaneous performance of multiple diagnostic modalities, including MSGB in this study. Second, RA patients were not randomly selected for this study, but were recruited only when they complained of sicca symptoms, which raises concerns about selection bias; the real prevalence of sSS in RA patients may be overestimated in this regard. Third, since we conducted tissue biopsy in minor salivary glands, but not major salivary glands, to diagnose SS, our histopathologic data may have limitations in direct comparison with SGUS and SWE data, which were all acquired in major salivary glands.

Despite the several limitations above, strong points also exist. This is the first study to perform full assessments that are potentially related to SS pathologies in sSS-suspected patients. Furthermore, to the best of our knowledge, newly introduced imaging modalities, such as SGUS and SWE, were first evaluated in this study in respect of their diagnostic performance and relationships with clinicopathologic features in sSS. The findings of the present study can support the diagnostic utility of SGUS in detecting sSS in RA patients, and the good correlations of SWE with histopathologic chronicity in both pSS and sSS. Taken together, the results of our work demonstrate that SGUS and SWE may be useful for assessing the presence of SS and the pathologic severity of fibrosis in sSS with RA patients, respectively, discriminating just sicca symptoms with no SS.

## Data Availability

The datasets used and/or analysed during the current study available from the corresponding author on reasonable request.
